# Novel Polyhydroxybutyrate-Degrading Activity of the *Microbulbifer* Genus as Confirmed by *Microbulbifer* sp. SOL03 from the Marine Environment

**DOI:** 10.4014/jmb.2109.09005

**Published:** 2021-10-27

**Authors:** Sol Lee Park, Jang Yeon Cho, Su Hyun Kim, Hong-Ju Lee, Sang Hyun Kim, Min Ju Suh, Sion Ham, Shashi Kant Bhatia, Ranjit Gurav, See-Hyoung Park, Kyungmoon Park, Yun-Gon Kim, Yung-Hun Yang

**Affiliations:** 1 Department of Biological Engineering, College of Engineering, Konkuk University, Seoul 05029, Republic of Korea; 2 Institute for Ubiquitous Information Technology and Applications, Konkuk University, Seoul 05029, Republic of Korea; 3 Department of Biological and Chemical Engineering, Hongik University, Sejong City 30016, Republic of Korea; 4 Department of Chemical Engineering, Soongsil University, Seoul 06978, Republic of Korea

**Keywords:** Poly(3-hydroxybutyrate), bioplastics, biodegradation

## Abstract

Ever since bioplastics were globally introduced to a wide range of industries, the disposal of used products made with bioplastics has become an issue inseparable from their application. Unlike petroleum-based plastics, bioplastics can be completely decomposed into water and carbon dioxide by microorganisms in a relatively short time, which is an advantage. However, there is little information on the specific degraders and accelerating factors for biodegradation. To elucidate a new strain for biodegrading poly-3-hydroxybutyrate (PHB), we screened out one PHB-degrading bacterium, *Microbulbifer* sp. SOL03, which is the first reported strain from the *Microbulbifer* genus to show PHB degradation activity, although *Microbulbifer* species are known to be complex carbohydrate degraders found in high-salt environments. In this study, we evaluated its biodegradability using solid- and liquid-based methods in addition to examining the changes in physical properties throughout the biodegradation process. Furthermore, we established the optimal conditions for biodegradation with respect to temperature, salt concentration, and additional carbon and nitrogen sources; accordingly, a temperature of 37°C with the addition of 3% NaCl without additional carbon sources, was determined to be optimal. In summary, we found that *Microbulbifer* sp. SOL03 showed a PHB degradation yield of almost 97% after 10 days. To the best of our knowledge, this is the first study to investigate the potent bioplastic degradation activity of *Microbulbifer* sp., and we believe that it can contribute to the development of bioplastics from application to disposal.

## Introduction

Poly(3-hydroxybutyrate) (PHB) is a well-studied bioplastic that can be produced from renewable biomass with physical properties similar to those of petroleum-based plastics [1]. Since PHB accumulates inside microorganisms as a carbon and energy storage compound under unfavorable growth conditions, it can also be degraded into water-soluble monomers, water, and carbon dioxide under carbon- and nitrogen-limiting conditions by microorganisms in a relatively short time compared to that needed for petroleum-based plastics [2]. As the biodegradability of PHB becomes more highly regarded as a great advantage in the industrial field, its replacement of petroleum-based plastics has been increasing.

There are several well-known PHB-degrading bacteria that utilize the mechanism of depolymerization for PHB degradation, including *Pseudomonas pickettii*, *Comamonas testosterone*, *Alcaligenes faecalis*, and *Cupriavidus necator* [3-6]. However, there are many undiscovered bacteria that can decompose PHB; thus, isolation and application of PHB-degrading bacteria in the disposal of bioplastics is regarded as an effective method of addressing bioplastic wastes and has led to additional studies [7, 8].

Members of the genus *Microbulbifer* are gram-negative Gammaproteobacteria found in high-salt environments, with blebs or vesicles on the surface of the cell [9]. There are few species in the *Microbulbifer* genus that are known as producers of bioactive substances as well as degraders of polysaccharides (Table 1). For example, *Microbulbifer* sp. 127CP7-12 can produce violacein, a natural purple pigment with antibiotic properties [10]. Additionally, some anticancer antibiotics, such as pelagiomicin or alkyl quinolones, are produced by *Microbulbifer* sp. Conversely, the *Microbulbifer* genus is well known for its hydrolytic activity toward polysaccharides, such as alginate, cellulose, agarose, and many other complex carbohydrates, with a considerable number of strains isolated from seaweed and its wastes. For example, *Microbulbifer elongatus* sp. HZ11 has been found to produce alginate lyase, and *Microbulbifer hydrolyticus* sp. IRE-31T is one of the most famous cellulase-producing strains [9, 11]; however, detailed gene information is still lacking. In addition, some strains are known to degrade agarose, especially β-agarose, thus playing a critical role in decomposing marine wastes. Although many species have been identified by their hydrolytic enzymes, to the best of our knowledge, the PHB degradation activity of the *Microbulbifer* genus has never been reported.

In this study, we isolated PHB-degrading strains from various soil samples and selected one of them, *Microbulbifer* sp. SOL03, which showed a strong biodegradation activity. We aimed to identify its biodegradability by measuring the degradation yield (%) with the residual PHB (mg) and establish the optimal conditions for the degradation of PHB with respect to temperature and salt concentration. In addition, we investigated the effects of various carbon and nitrogen sources on biodegradation. Finally, we cultured the isolate on solid plates containing other bioplastics to assess its biodegradability toward other bioplastics as well.

## Materials and Methods

### Chemicals

All chemicals used in this study were of analytical grade. Solvents used in making plates and film such as chloroform and dichloromethane (DCM) were obtained from Sigma-Aldrich (USA). Sodium dodecyl sulfate (SDS) was purchased from Biosesang (Korea). PHB pellets were obtained from Goodfellow (UK) and other bioplastic pellets and sodium 3-hydroxybutyrate (3HB) were purchased from Sigma-Aldrich. Carbon and nitrogen sources were purchased from Junsei (Japan).

### 16S rRNA Sequencing

Colonies forming clear zones on the PHB plates were identified at the species level using 16S rRNA sequencing by PCR amplification using the primer 27F [12]. Partial sequences were obtained by Bionics (Korea) and compared to those in the NCBI GenBank database (https://blast.ncbi.nlm.nih.gov/Blast.cgi) using BLASTN tools [13].

### Preparation of Bioplastic-Containing Medium

For the preparation of the media plates containing other bioplastics (polylactic acid; PLA, polybutylene succinate; PBS, polybutylene adipate terephthalate; PBAT, polycaprolactone; PCL), 0.2 g of commercial bioplastic pellets were dissolved in 40 ml of DCM in a 60°C water bath until they were dissolved. After the bioplastics were dissolved in DCM, SDS and distilled water were added, and the mixture was sonicated for 10 min using a Vibra-Cell VCX500 by Sonics & Materials, Inc. (USA) with 15 s of pulse and an amplitude of 40% to mix solvent phase and water phase uniformly, resulting in an opaque emulsion [14]. After sonication, the solvent was completely evaporated in a fume hood using a stirrer set at 60°C, so that the cells could grow without any toxicity. After evaporation, marine broth (MB) containing peptone (5.0 g/l), yeast extract (1.0 g/l), ferric citrate (0.1 g/l), sodium chloride (19.45 g/l), magnesium chloride (5.9 g/l), magnesium sulfate (3.24 g/l), calcium chloride (1.8 g/l), potassium chloride (0.55 g/l), sodium bicarbonate (0.16 g/l), potassium bromide (0.08 g/l), strontium chloride (34.0 mg/l), boric acid (22.0 mg/l), sodium silicate (4.0 mg/l), sodium fluoride (2.4 mg/l), ammonium nitrate (1.6 mg/l), disodium phosphate (8.0 mg/l) and agarose were added and autoclaved [13, 15].

### PHB Degradation Assays

The conventional solvent-cast method was used to prepare PHB films. One gram of PHB pellets was completely emulsified in 100 ml of chloroform for 16 h at 60°C. The solvent was evaporated in a fume hood until a plastic film formed. Then, it was cut into small pieces weighing 20 mg each. The prepared PHB film (20 mg) was autoclaved at 12°C for 15 min and cultured in 5 ml of liquid MB with the isolated *Microbulbifer* sp. SOL03 at 30°C for 10 days. Cultivation was performed in a rotary shaker at 200 rpm. The residual PHB films were collected, washed with distilled water, and freeze-dried for preparation of the GC sample. In addition to the liquid assay, PHB degradability was confirmed using a solid-based method. Paper discs by Toyo Roshi Kaisha (Japan) were placed in the middle of the PHB plate, inoculated with 10 μl of the culture medium, and incubated at 30°C. Then, the radius of the clear zone formed on the plate was measured [15, 16].

### Gas Chromatography

The residual PHB quantity was determined using gas chromatography (GC) as previously described, with slight modifications [17-20]. For analysis, the culture medium was centrifuged at 10,000 ×*g* for 10 min to collect the residual PHB films, followed by a washing process with deionized water to remove cell residue attached to the film. The residual PHB films were lyophilized in Teflon-stoppered glass vials. For methanolysis of the PHB samples, 1 ml of chloroform and 1 ml of a methanol/sulfuric acid (85:15 v/v) mixture was added to the vials and incubated at 100°C for 2 h. The samples were then cooled at 25°C for 10 min and 1 ml of ice-cold water was added. Then, the samples were mixed thoroughly and the organic phase was extracted using a pipette and transferred to clean borosilicate glass tubes containing anhydrous sodium sulfate. The samples were then injected into a GC instrument by Young Lin Tech (Korea) using an HP-FFAP column (30 m × 0.32 mm × 0.25 μm) by Agilent Technologies (USA). The split ratio was 1:10. Helium was used as the carrier gas, and the flow rate was maintained at 3.0 ml/min. A 2-μl portion of the organic phase was injected using an autosampler. The inlet temperature was kept at 210°C. The column oven was held at 80°C for 5 min, heated to 220°C at 20°C/min, and then held at 220°C for another 5 min. Peak detection was performed using a flame ionization detector maintained at 230°C [21].

### Analysis of Physical Properties

To observe the changes in the surface of the PHB film after degradation, scanning electron microscopy (SEM) was performed. For SEM analysis, the residual PHB films from each day were collected by centrifugation, washed three times with phosphate buffer (pH 6.8–7.0), and fixed with 2% buffered glutaraldehyde overnight. Glutaraldehyde was decanted after centrifugation and the samples were washed with phosphate buffer to get rid of the residual glutaraldehyde. The samples were dehydrated using a gradually increasing concentration of ethanol (50%, 70%, 95%, and 100%). For chemical drying, different ratios of ethanol and hexamethyldisilazane (HMDS; 2:1, 1:1, 1:2 v/v) were used; 100% HMDS was used in the final step, and the mixture of HMDS and sample was mounted on specimen stubs. HMDS was evaporated overnight in a fume hood. The samples were then coated with gold dust at 5 mA for 120 s, and back-scatter electron images were acquired using a scanning electron microscope TM4000Plus by Hitachi, Ltd. (Japan) at an accelerating voltage of 5–15 kV [22].

The differences in the functional groups of the PHB film were detected using Fourier-transform infrared spectroscopy by Nicolet 6700; Thermo Fisher Scientific (USA) in the scanning range of 4,000 to 600 cm^-1^. The resolution was set to 4 cm^-1^, and 32 scans were recorded for each spectrum with an auto base [23, 24]. The residual PHB film was washed with distilled water and lyophilized for analysis.

Gel permeation chromatography by Youngin Chromass (Korea) was performed to determine the molecular weight and molecular mass distribution of PHB. For sample preparation, the PHB pellet was dissolved in chloroform and stirred constantly at 700 rpm using a thermo-shaker at 60°C for 2 h. After dissolving the PHB film, ice-cold water was added at three times the volume of the dissolved PHB solution and mixed thoroughly. Then, precipitated solvent phase was collected and the solvent was evaporated again. Finally, it was dissolved in the chloroform again, resulting in easily dissolved PHB sample with impurities in the polymer removed. This solution was filtered through a syringe filter by Chromdisc (Korea) with a pore size of 0.2 μm to separate the dissolved PHB from the remaining insoluble cell components. A high-performance liquid chromatography (HPLC) apparatus consisting of a loop injector (Rheodyne 7725i; Sigma-Aldrich), an isocratic pump with dual heads (YL9112), a column oven (YL9131), columns (Shodex, K-805, 8.0 I.D. × 300 mm, Shodex, K-804, 8.0 mm I.D. × 300 mm; Showa Denko K.K., Japan), and a refractive index detector (YL9170) was used for analysis. A total of 60 μl of the solution without air bubbles was injected. Chloroform was used as the mobile phase at a flow rate of 1 mL/min and a temperature of 35°C. The data were analyzed using YL-Clarity software for a single YL HPLC instrument by Youngin Chromass. The molecular masses were analyzed relative to polystyrene standards ranging from 5,000 to 2,000,000 g/mol [25, 26].

## Results and Discussion

### Isolation of PHB-Degrading Bacteria and Biodegradability Identification

**Fig. 1:**
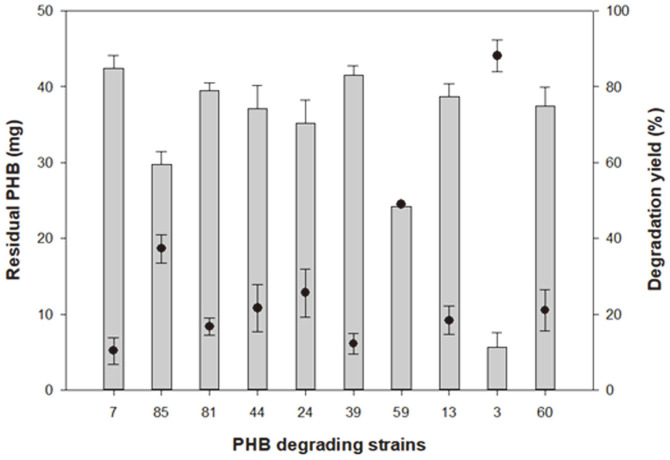
Polyhydroxybutyrate (PHB) degradation yield of 10 isolated stains. The residual PHB (mg) measured by gas chromatography is presented on the left axis and the degradation yield (%) calculated by comparing the residual PHB (mg) to the original amount of PHB is presented on the right axis.

**Fig. 2:**
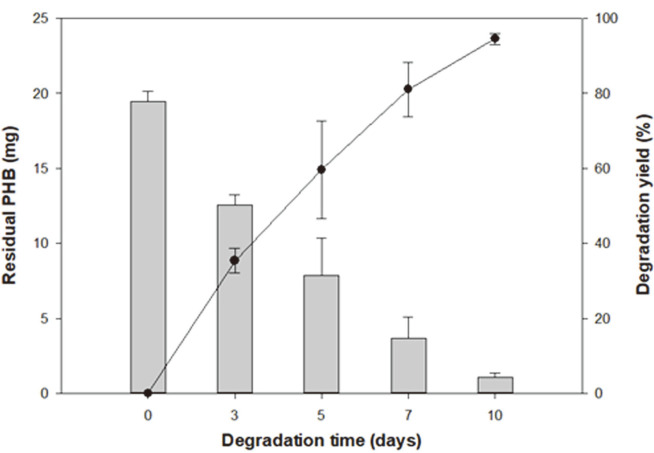
Degradation yield (%) of *Microbulbifer* sp. SOL03 measured over 10 days. *Microbulbifer* sp. SOL03 cells were cultured in a liquid marine broth (MB) medium with a polyhydroxybutyrate (PHB) film at 30°C for 10 days. The residual PHB (mg) measured by gas chromatography is presented on the left axis and the degradation yield (%) calculated by comparing the residual PHB (mg) to the original amount of PHB is presented on the right axis.

PHB-degrading bacteria were isolated from soil samples collected at a depth of 0–10 cm from the surface of various seashores in Korea [15]. As a result, 13 strains were isolated, of which 10 were tested for PHB degradation activity. They were cultured in liquid MB with 20 mg of PHB film prepared using the solvent-cast method [22]. The residual PHB (mg) was measured by GC, and the degradation yield (%) was calculated relative to the original amount of PHB (mg). Ten isolates, namely *Bacillus* sp. SOL07 (96.96% 16S rRNA similarity to *Bacillus thioparans*), *Bacillus* sp. SOL13 (80.01% 16S rRNA similarity to *Bacillus infantis*), *Microbulbifer* sp. SOL03 (95.81% 16S rRNA similarity to *Microbulbifer taiwanensis*), *Bacillus* sp. SOL24 (96.44% 16S rRNA similarity to *Bacillus aquimaris*), *Bacillus* sp. SOL39 (94.10% 16S rRNA similarity to *Bacillus pakistanensis*), *Bacillus* sp. SOL44 (95.94% 16S rRNA similarity to *Bacillus aryabhattai*), *Bacillus* sp. SOL59 (97.89% 16S rRNA similarity to *Bacillus subterraneus*), *Bacillus* sp. SOL60 (97.83% 16S rRNA similarity to *Bacillus zanthoxyli*), *Bacillus* sp. SOL81 (95.09% 16S rRNA similarity to *Bacillus hwajinpoensis*), and *Bacillus* sp. SOL85 (98.84% 16S rRNA similarity to *Bacillus megaterium*), were cultured for 5 days at 30°C (Fig. 1, Table 2). Most strains showed approximately 20% degradation yield, while *Bacillus* sp. SOL85, *Bacillus* sp. SOL59, and *Microbulbifer* sp. SOL03 showed a high degradation yield. In particular, *Microbulbifer* sp. SOL03 showed the highest degradation yield at more than 80% after five days. Therefore, for detailed experiments concerning the biodegradation of PHB, *Microbulbifer* sp. SOL03 was selected because of its high biodegradation activity. To identify its degradation activity, it was cultured in a liquid MB medium with 20 mg of PHB film at 30°C for 10 days (Fig. 2). The residual PHB (mg) after 3, 5, 7, and 10 days was collected and measured by GC, and the corresponding degradation yield (%) was calculated. As a result, the degradation yield gradually increased over 10 days, showing approximately 40% degradation yield in the first 3 days, and it steadily degraded the PHB film over the remaining days, reaching about 80% degradation yield after 7 days. Finally, it showed a degradation yield of approximately 97% on the final day, leaving only minute particles of the PHB film. In addition to the degradation yield measured using the liquid-based method, *Microbulbifer* sp. SOL03 was grown on solid MB medium with emulsified PHB to observe clear zone formation. However, it grew much slower on the solid medium than on the liquid medium, so that the clear zone formed only on the surface of the colony after 10 days, with a radius less than 1 mm (data not shown).

### Optimal Temperature and Salt Concentration for PHB Degradation

**Fig. 3:**
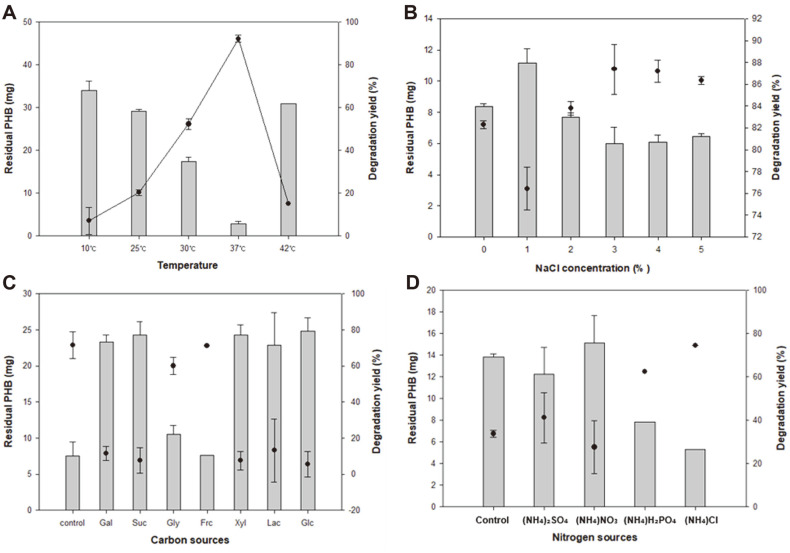
Optimal conditions for polyhydroxybutyrate (PHB) degradation concerning temperature, NaCl concentration, the effect of the additional carbon and nitrogen sources. (**A**) *Microbulbifer* sp. SOL03 cells were cultured at various temperatures ranging from 10°C to 42°C. (**B**) *Microbulbifer* sp. SOL03 cells were cultured with the addition of various concentrations of NaCl ranging from 0% to 5%. (**C**) Various carbon sources were added, including galactose (Gal), sucrose (Suc), glycerol (Gly), fructose (Frc), xylose (Xyl), lactose (Lac), and glucose (Glc). (**D**) Nitrogen sources were added, including ammonium sulfate [(NH_4_)_2_SO_4_], ammonium nitrate [(NH_4_)NO_3_], ammonium dihydrogen phosphate [(NH_4_)H_2_PO_4_], and ammonium chloride [(NH_4_)Cl].

Although *Microbulbifer* sp. SOL03 showed a high degradation activity even under general conditions in liquid media, the optimal temperature and salt concentration were established for improved biodegradation activity. *Microbulbifer* sp. SOL03 cells were cultured in liquid MB medium with PHB film at various temperatures ranging from 10°C to 42°C for 4 days, and the residual PHB (mg) was measured using GC (Fig. 3A). The results showed that the residual PHB (mg) gradually decreased as the temperature increased to 37°C, reaching a maximum degradation yield (%) of approximately 97% at 37°C. However, when the temperature rose to 42°C, it rarely degraded the PHB film, showing an almost similar degradation yield (%) to that at 25°C. This might be attributed to the susceptibility of PHB to PHB depolymerase at high temperatures in the range required for cell growth [27]. In other words, the solubility of PHB increased as the temperature increased, so that it was attacked more easily by the depolymerase at 37°C than at 30°C, showing a higher degradation yield at 37°C. However, cells rarely grew at temperatures over 42°C, so the degradation yield significantly decreased regardless of the solubility of the PHB, and the culture showed poor degradation yield [28, 29].

In addition to the optimal temperature, the optimal salt concentration was determined by adjusting the salt concentration from 0% to 5% (w/v) with additional NaCl in a liquid MB medium, and the *Microbulbifer* sp. SOL03 cells were cultured with the PHB film at 30°C (Fig. 3B). The results showed a high degradation yield (%) with 3%NaCl, representing an approximately 88% degradation of the PHB film, while a decreasing tendency of degradation yield (%) was obtained when more than 4% of NaCl was added, with the higher salt concentration probably inducing salt stress and negatively affecting the degradation potential of the cell.

### Effect of Carbon and Nitrogen Sources in Biodegradation

To determine the effect of carbon and nitrogen sources on the biodegradation of the PHB film, different carbon and nitrogen sources were added to the culture medium of *Microbulbifer* sp. SOL03 grown in the presence of a PHB film at 30°C for 4 days. For the carbon sources, galactose, sucrose, glycerol, fructose, xylose, lactose, and glucose were added at 1% (w/v) concentration, and the residual PHB films were compared to the control group cultured in the absence of carbon sources (Fig. 3C). The results showed a maximum degradation yield (%) of approximately 70% in the absence of any carbon sources. In addition, decent degradation activity was observed when glycerol and fructose were added, showing a similar or slightly lower degradation yield than that of the control. This phenomenon seems to be attributed to carbon catabolite repression, commonly observed in PHA-degrading organisms, suggesting that cells preferentially utilize a favorable carbon source other than PHB, thereby decreasing the PHB degradation activity of the cell [3, 30, 31]. In this case, the cell could utilize additional carbon sources, except for glycerol and fructose, prior to PHB, resulting in the repression of PHB depolymerase expression. Consequently, other carbon sources, apart from glycerol and fructose, deteriorated the degradation activity of the strain, showing less than 10% degradation yield, which was remarkably inferior to that of the control, and with most of the PHB films left intact.

In addition to the carbon sources, four types of nitrogen sources, 0.1% (w/v) of ammonium sulfate [(NH_4_)_2_SO_4_], ammonium nitrate [NH_4_NO_3_], ammonium dihydrogen phosphate [(NH_4_)H_2_PO_4_], and ammonium chloride [NH_4_Cl], were added to the culture medium of *Microbulbifer* sp. SOL03 to determine the effect of nitrogen sources on the biodegradation of PHB (Fig. 3D). The results showed a maximum degradation yield (%) of approximately 78% when ammonium chloride was added, followed by the addition of ammonium dihydrogen phosphate, showing an almost 62% degradation yield. Since nitrogen sources are known to influence the production and secretion of enzymes, the addition of ammonium chloride might support the synthesis of enzymes, including PHB depolymerase, in vivo, resulting in improvement in the biodegradation of PHB [32].

### Physical Properties of PHB after Biodegradation

**Fig. 4:**
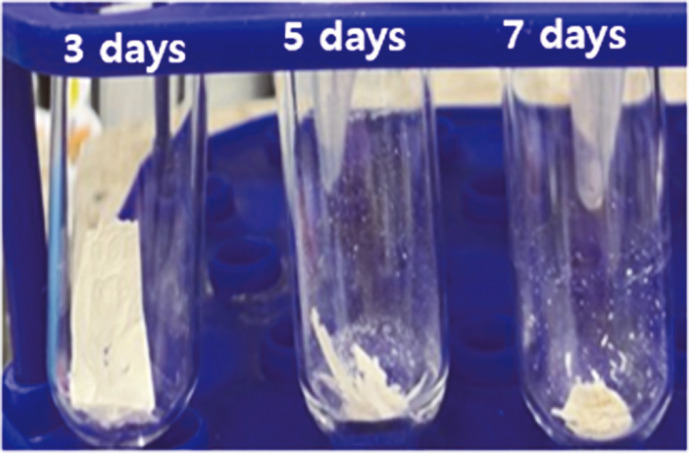
Residual polyhydroxybutyrate film collected and lyophilized for preparation of gel permeation chromatography analysis. The samples were collected on days 3, 5, and 7.

Biodegradation of PHB is accompanied by changes in its physical properties, such as molecular weight, surface morphology, and functional groups. The molecular weights (Mw) of the degraded PHB films were analyzed by gel permeation chromatography (GPC) and compared with the original molecular weight of the PHB film (Table 3). The PHB films were collected from the culture medium and washed with distilled water until the cell biomass attached to the PHB film was removed. The collected samples were lyophilized, and the residual PHB films were compared (Fig. 4). It seemed clear that the original PHB film was cut into many small pieces as degradation progressed. To prepare samples for GPC analysis, the residual PHB films were completely emulsified in chloroform at 60°C and filtered through a syringe filter with a pore size of 0.2 μm. According to the Mw data of the samples, the intact PHB film showed a Mw of 587 × 10^3^ with a polydispersity index (PDI) of 1.3, indicating low dispersity of the molecular weight of the PHB film. As the degradation progressed, the molecular weight of the degraded PHB film decreased to a molecular weight of 475 × 10^3^ on day 3, followed by 102 × 10^3^ on day 5, and showed a molecular weight of 41 × 10^3^ on the final day. Unlike the gradual decrease in molecular weight throughout the biodegradation process, the PDI increased to 10.4 during the first 3 days. This phenomenon seemed to be due to the presence of many oligomers with various molecular weights, resulting from the biodegradation of PHB. With a further progression of the biodegradation of PHB, the PDI steadily decreased and converged to 1.7 on the final day, suggesting that by this time, most of the degraded PHB particles had low molecular weight, causing the PDI to return to a low index.

**Fig. 5:**
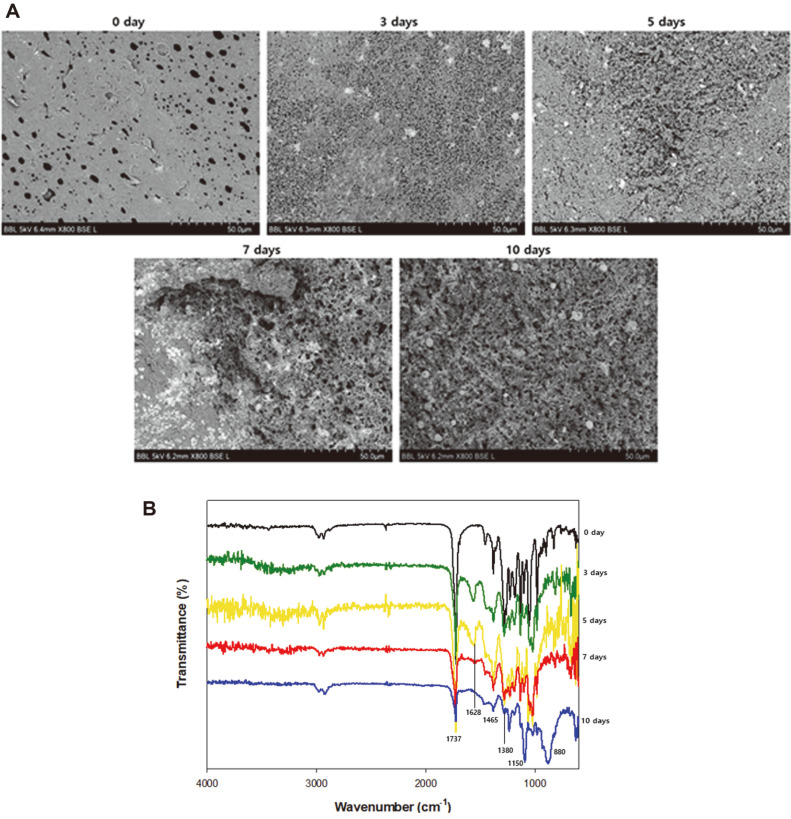
Physical properties of degraded polyhydroxybutyrate (PHB) films. (**A**) Surface changes of PHB films cultured with *Microbulbifer* sp. SOL03 in liquid medium for 10 days monitored by scanning electron microscopy. (**B**) Fouriertransform infrared data of PHB film before and after degradation.

In addition, changes in the surface of the PHB film were observed using SEM throughout the biodegradation process. Since biodegradation begins with the secretion of depolymerases that adhere to hydrophobic PHB surfaces and accelerate surface erosion, observation of surface changes is necessary [4, 33, 34]. *Microbulbifer* sp. SOL03 cells were cultured in liquid MB medium with a PHB film at 30°C for 10 days. Each day, the PHB films were collected and washed with distilled water. To obtain clearer images, the samples were coated with gold to prevent edge effects [35]. The samples were observed at 800 × magnification, which was suitable for observing the overall surface changes (Fig. 5A). The intact PHB film had a smooth and even surface, and as biodegradation progressed, the surface became partially rough and uneven with large cracks on it. Finally, the whole surface became rough, with numerous large pores appearing on the surface, while only a small part of the surface remained intact due to surface erosion by the depolymerase secreted by *Microbulbifer* sp. SOL03.

Finally, the degraded PHB films were analyzed by Fourier-transform infrared spectroscopy (FT-IR) to detect changes in the functional groups after biodegradation. *Microbulbifer* sp. SOL03 cells were cultured in liquid MB medium with a PHB film at 30°C for 10 days. The residual PHB films were collected, washed with distilled water, and lyophilized. The FT-IR analysis of each sample showed differences in intensity at certain wavenumber points, indicating changes in their functional groups after biodegradation (Fig. 5B). The spectra exhibited some weak peaks with consistent intensity between 2,000 and 3,000 cm^-1^, representing the C-H stretching bond [27]. However, at some wavenumbers, the intensity of the peaks changed as the degradation progressed. For example, the peaks at 880 cm^-1^ (C-H bending), 1,380 cm^-1^ (C-H bending), 1,465 cm^-1^ (C-H bending), 1,628 cm^-1^ (C=O stretching) and 1,737 cm^-1^ (C=O stretching) exhibited some changes in transmittance as the degradation progressed, indicating that these chemical bonds were affected by *Microbulbifer* sp. SOL03 as it grew and degraded the film. In particular, the peak at 1,737 cm^-1^ decreased, exhibiting the highest transmittance in the intact PHB film and decreased transmittance in the degraded PHB films over 10 days. This indicates that the ester bonds, which connect monomers of PHB together, are cleaved throughout the biodegradation process, resulting from the biodegradation activity of *Microbulbifer* sp. SOL03.

### Growth of SOL03 with Additional 3-Hydroxybutyrate (3HB)

**Fig. 6:**
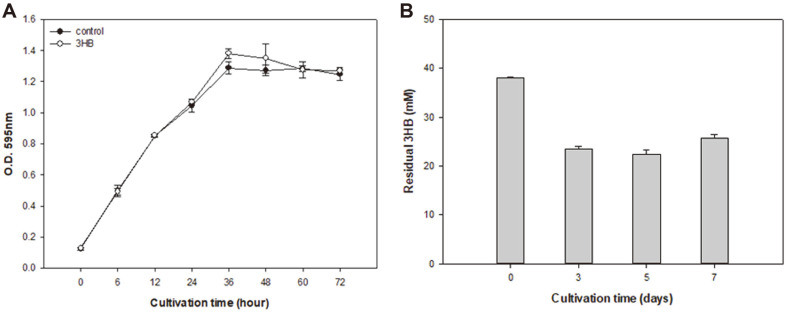
*Microbulbifer* sp. SOL03 cells cultured with 3-hydroxybutyrate (3HB). (**A**) Growth curve of the *Microbulbifer* sp. SOL03 cells in the presence of 3HB. (**B**) Residual 3HB (mM) measured by liquid chromatography is presented on the left axis and the consumption rate (%) compared to the original amount of 3HB is presented on the right axis.

As *Microbulbifer* sp. SOL03 was confirmed as a PHB-degrading bacterium, the monomer of PHB was supplied as a carbon source instead of PHB, to determine whether *Microbulbifer* sp. SOL03 can utilize the monomer form of PHB along with the polymer form. *Microbulbifer* sp. SOL03 cells were cultured in liquid MB medium with water-soluble 3HB for 7 days at 30°C, and the growth rate was measured by determining the optical density at 595 nm (Fig. 6A). The cells showed a lag phase during the early 4 h, but then entered the exponential phase until 36 h, producing the highest cell density. At this point, although the MB medium contained 0.5% peptone and 0.1% yeast extract, the cells grew better when 0.5% 3HB was supplied, representing higher cell density than that of the control, indicating that it could not only utilize the monomer form of PHB but also that the cell growth was affected in a positive way by the addition of 3HB. After the cell density remained steady until 48 h, it started to decrease, due to cell death caused by the accumulation of the toxic by-products of cellular metabolism. In addition, the residual 3HB (mM) after cultivation was measured using liquid chromatography (Fig. 6B). The amount of 3HB (mM) supplied was the same as that of PHB, and the supernatant of the culture medium was collected after centrifugation and diluted 10-fold. As a result, the amount of 3HB reduced until 5 days, showing 40% of the consumption rate, with the fastest decrease observed during the first 3 days, suggesting that fast cell growth during the early 36 h might accelerate enzyme activity. However, it showed a decreased consumption rate of approximately 30% after 5 days, which might be related to the induction of the death phase after 48 h. This shows that the cells that have grown using a carbon source in the medium can then secrete depolymerase, resulting in a breakdown of the PHB into its monomer 3HB, which the cells can then take up and grow even when the carbon sources in the medium are exhausted, as indicated by the consumption rate of 3HB.

### Capacity for Biodegradation of Other Plastics

The biodegradation activity of *Microbulbifer* sp. SOL03 was tested on other bioplastics (Table 4). For the other bioplastics, flexible aliphatic polyesters, such as PLA, PBS, PBAT, PCL, and poly(3-hydroxybutyrate-*co*-4-hydroxybutyrate) [P(3HB-*co*-4HB)], were used in the experiments. The bioplastics were emulsified in MB agar medium, and *Microbulbifer* sp. SOL03 cells were cultured on the plates for 3 days at 30°C. Following incubation, clear zones were observed on the plates. The *Microbulbifer* sp. SOL03 could not only degrade PHB but also P(57 mol% 3HB-co-43 mol% 4HB), P(88 mol% 3HB-co-12 mol% 3HV), and PCL. Although the clear zone on the PCL plate was not as large as that on the copolymer plates, it was clear that it could degrade PCL as well. This might be because the copolymer has a porous surface with low crystallinity compared with that of the homopolymer, which makes the biodegradation of copolymers easier [36, 37].

As the applications for bioplastics have increased, the issue of biodegradation has also gained weight. In particular, PHB is one of the most commercialized bioplastics that can be produced or decomposed by microorganisms, making it highly eco-friendly. Therefore, the disposal of bioplastics using microbial decomposers is attracting the interest of the public. In this respect, we established a suitable method for preparing media plates for the screening of PHB-degrading bacteria and for carrying out the characterization process in a previous study, resulting in numerous PHB-degrading isolates. We examined their degradation activity by comparing the residual PHB (mg) with the original amount of PHB and selected the strain with the greatest degradation activity, *Microbulbifer* sp. SOL03. We then measured the degradation yield by culturing the strain in liquid medium with a PHB film for 10 days; the strain showed a degradation yield of 97%. The physical properties of the degraded PHB films were compared to those of the intact PHB film with respect to surface change, molecular weight, and functional groups. Finally, we applied *Microbulbifer* sp. SOL03 to other bioplastics and confirmed that it could degrade not only copolymers, including P(3HB-co-4HB) and P(3HB-co-HV), but also PCL. We are not presently aware of why *Microbulbifer* sp. SOL03 showed a significant difference in biodegradation between the solid and liquid media; however, to the best of our knowledge, this is the first report on the biodegradability of a *Microbulbifer* sp., which can completely degrade PHB within 10 days in liquid media.

## Figures and Tables

**Table 1:** List of *Microbulbifer* sp. reported as producers or degraders of various compounds.

Roles	Categories	Target compound	Enzymes found	Species	References
1) Producer	Pigment	Violacein	VioABCDE	*Microbulbifer* sp. 127CP7-12	[38]
	Antibiotics	Pelagiomicin	-	*Microbulbifer variabilis* sp. Ni-2088T	[10]
		Alkyl quinolones	PqsABCDEH	*Microbulbifer elongatus* sp. HZ11	[11]
2) Degrader	Polysaccharides	Alginate	AlgMsp	*Microbulbifer harenosus* sp. JCM 32688T	[39]
			-	*Microbulbifer mangrovi* sp. KCTC 23483T	[40]
			Aly	*Microbulbifer elongatus* sp. HZ11	[41]
		Cellulose	-	*Microbulbifer epialgicus* sp. GL-2	[42]
			-	*Microbulbifer hydrolyticus* sp. IRE-31T	[9]
		β-Agarose	MtAgaA	*Microbulbifer thermotolerans* sp. DSM 19200T	[43]
			-	*Microbulbifer agarilyticus* sp. DSM 19189T	[44]
	Polyesters	PHB	-	*Microbulbifer* sp. SOL03	In this study

**Table 2:** Screened strains.

Isolates	Related strains	Identity	Isolated temperature
SOL03	*Microbulbifer taiwanensis*	95.81%	30°C
SOL06	*Bacillus trueperi*	97.06%	30°C
SOL07	*Bacillus thioparans*	96.96%	30°C
SOL13	*Bacillus infantis*	80.01%	30°C
SOL24	*Bacillus aquimaris*	96.44%	30°C
SOL39	*Bacillus pakistanensis*	94.01%	30°C
SOL44	*Bacillus aryabhattai*	95.94%	30°C
SOL59	*Bacillus subterraneus*	97.89%	37°C
SOL60	*Bacillus zanthoxyli*	97.83%	37°C
SOL61	*Halobacillus kuroshimensis*	97.01%	37°C
SOL81	*Bacillus hwajinpoensis*	95.09%	30°C
SOL85	*Bacillus megaterium*	98.84%	30°C
SOL88	*Lysinibacillus dysseyi*	89.34%	30°C

**Table 3:** Molecular weight change of the PHB film throughout biodegradation.

	M_w_ (×10^3^)	M_n_ (×10^3^)	Mw/Mn
Control	587	438	1.3
3 days	476	46	10.4
5 days	103	29	3.6
7 days	42	25	1.7

*weight-average molecular weight (Mw) and number-average molecular weight (Mn)

**Table 4:** Clear zone formation on plates containing other bioplastics.

	P(3HB-co-4HB)	P(3HB-co-HV)	PCL	PBS	PBAT	PLA
*Microbulbifer* sp. SOL03	+	+	+	-	-	-
